# Balance of Active, Passive, and Anatomical Cardiac Properties in Doxorubicin-Induced Heart Failure

**DOI:** 10.1016/j.bpj.2019.07.033

**Published:** 2019-07-29

**Authors:** Alexandre Lewalle, Sander Land, Jort J. Merken, Anne Raafs, Pilar Sepúlveda, Stéphane Heymans, Jos Kleinjans, Steven A. Niederer

**Affiliations:** 1 Department of Biomedical Engineering, St Thomas’s Hospital, King’s College London, London, United Kingdom; 2 Department of Cardiology, Maastricht University, Maastricht, the Netherlands; 3 Regenerative Medicine and Heart Transplantation Unit, Instituto de Investigación Sanitaria La Fe, Valencia, Spain; 4 Department of Toxicogenomics, Maastricht University, Maastricht, the Netherlands

## Abstract

Late-onset heart failure (HF) is a known side effect of doxorubicin chemotherapy. Typically, patients are diagnosed when already at an irreversible stage of HF, which allows few or no treatment options. Identifying the causes of compromised cardiac function in this patient group may improve early patient diagnosis and support treatment selection. To link doxorubicin-induced changes in cardiac cellular and tissue mechanical properties to overall cardiac function, we apply a multiscale biophysical biomechanics model of the heart to measure the plausibility of changes in model parameters representing the passive, active, or anatomical properties of the left ventricle for reproducing measured patient phenotypes. We create representative models of healthy controls (*N* = 10) and patients with HF induced by (*N* = 22) or unrelated to (*N* = 25) doxorubicin therapy. The model predicts that HF in the absence of doxorubicin is characterized by a 2- to 3-fold stiffness increase, decreased tension (0–20%), and ventricular dilation (of order 10–30%). HF due to doxorubicin was similar but showed stronger bias toward reduced active contraction (10–30%) and less dilation (0–20%). We find that changes in active, passive, and anatomical properties all play a role in doxorubicin-induced cardiotoxicity phenotypes. Differences in parameter changes between patient groups are consistent with doxorubicin cardiotoxicity having a greater dependence on reduced cellular contraction and less anatomical remodeling than HF not caused by doxorubicin.

## Significance

Doxorubicin, a common chemotherapeutic drug, is a known cause of heart failure. The relative significance of its molecular, cellular, and tissue physiological effects in shaping the long-term condition remains unclear. Diagnosis and treatment options are hence severely limited, given the gap in understanding between symptoms and underlying causes. This study aims to bridge this gap through computational biomechanical modeling, linking left-ventricular mechanics with clinical measurements. By providing a framework for integrating and interpreting heart-failure clinical measurements (either associated with or independent of doxorubicin), we seek to identify underlying mechanisms specific to cardiotoxic heart failure. Ultimately, identifying these dominant mechanisms may help improve diagnosis, therapies, and at-risk patient identification in this challenging patient group.

## Introduction

Doxorubicin is a common anthracycline chemotherapeutic drug with multitudinous irreversible cardiotoxic side effects that increase the risk of heart failure (HF) (1, 2, 3). The incidence rate of congestive heart failure after anthracycline treatment is dose-dependent and has been shown to be as high as 9% (4, 5, 6). The study and clinical treatment of patients is inherently challenging given the timescales for the development of symptoms, which range from weeks to years after cessation of chemotherapy. Cardiotoxic effects are thus confounded with natural aging, lifestyle, and predisposition to cardiovascular disease. Cardiotoxicity patients are identified as having a reduced left-ventricular (LV) ejection fraction (LVEF, defined as the ratio of the blood volume ejected per heartbeat into the circulation heartbeat/the end-diastolic volume) (7). At this irreversible stage, there are limited to no treatment options available beyond conventional HF therapy. Identifying the changes in cardiac properties that give rise to the reduced cardiac function seen in these patients may facilitate the development of improved monitoring, early HF detection, and therapies tailored to the pathologies of this specific patient group.

The multifariousness of doxorubicin cardiotoxicity constitutes a major challenge for developing systematic treatment strategies. At the subcellular molecular level, doxorubicin facilitates the formation of free radicals and reactive oxygen species, thereby generating significant oxidative stress, particularly in mitochondria (8, 9). This affects a multitude of dependent pathways, resulting in interference with gene expression by inhibiting topoisomerases (10), the degradation of mitochondrial function (11, 12), cardiomyocyte apoptosis (13), intracellular calcium dysregulation (14, 15), myofibrillar deterioration (16), the deterioration of cardiomyocyte structure (1), and cardiac energy homeostasis generally (2). These cellular-level effects have consequences at a higher functional level. Doxorubicin increases the ability of tumor cells to disintegrate the extracellular matrix, thereby impairing tumor cell motility and the structural integrity of the cardiac tissue (17). Reduced LVEF suggests a deterioration in the contractile tension of the muscle tissue. Early cardiac fibrosis has also been observed in doxorubicin-treated patients with potential impact on the passive mechanical tissue properties (18). At the organ level, doxorubicin may also be associated with dilation of the LV cavity (19). Despite the abundance of research into all these aspects of cardiotoxicity, their relative importance in the overall development of HF is unknown. Thus, the clinical treatment of the ensuing HF generally focuses on alleviating symptoms independently of underlying causes. There is presently no systematic methodology for linking drug targets with long-term clinical outcomes in this patient group (20).

The aim of this study is to identify characteristic changes in cellular and material function, specific to this patient group, that could be used for developing early identification biomarkers or informing patient treatment. We propose to combine known physiology, inherent physical laws, and pressure, motion, and anatomical measurements within a single biophysical framework to generate plausible estimates of the dominant factors that give rise to cardiotoxic HF. We used computational simulations of the cardiac cycle to map principal clinical cardiac phenotypes routinely measured in HF patients (the LVEF, the LV end-diastolic diameter (LVEDD), and the maximal ejection pressure (MEP)) onto underlying mechanistic properties. To reproduce the observed phenotype variations with maximal objectivity while acknowledging the scale of the complexity of the physiological system, our analysis considered three broad classes of cardiac features from a phenomenological perspective: the strength of active contraction, passive mechanical properties, and anatomical dimensions of the LV. Model parameters expressing these properties were constrained using LVEF, LVEDD, and MEP measurements, as well as measurements of the collagen volume fraction (CVF) performed on biopsies. Hence, by separately analyzing data from healthy and diseased patients, we sought to determine 1) what alterations to mechanistic properties of healthy hearts can most plausibly account for the development of the HF phenotypes and 2) whether such alterations differ in HF due to doxorubicin compared to HF resulting from other causes. Identifying the dominant or even the most likely factors responsible for cardiotoxicity-induced HF is an important first step in developing future studies for at-risk patient identification, early diagnosis, and treatment.

## Materials and Methods

### Clinical characterization

#### Patient cohorts

All patients were recruited at the Academic Hospital Maastricht, Maastricht University Medical Centre in a study approved by the local ethics committee and adhering to the declaration of Helsinki. Three patient cohorts were considered:

Cardiotoxic HF patients (HF_C_) (Table S1, *N* = 22) were doxorubicin-treated cancer patients with HF symptoms (e.g., dyspnea, fatigue, edema, reduced LVEF). The HF symptoms developed typically 1 year or more after the cessation of chemotherapy (average 6 ± 6 years). HF treatments involved a range of medications and dosage adapted to each patient according to their body mass (Table S4). Average age = 55 ± 13 years, body mass index (BMI) = 26 ± 3 kg/m^2^, gender = 23% male, 77% female.

Noncardiotoxic HF patients (HF_0_) (Table S2, *N* = 25) were patients suffering from HF (same symptoms as for HF_C_) with no history of chemotherapy. Average age = 55 ± 14 years, BMI = 27 ± 5 kg/m^2^, gender 32% male, 68% female. HF treatments involved a range of medications and dosage adapted to each patient according to their body mass (Table S5).

Healthy adults (HA) (Table S3, *N* = 10) were average age = 49 ± 10, BMI = 27 ± 4, gender = 80% male, 20% female.

All the data reported in the manuscript were obtained when patients were admitted to the hospital and diagnosed with idiopathic HF (i.e., excluding ischemia or valvular disease as causes of HF). However, the cohorts were heterogeneous in the sense that some patients were already undergoing HF medical treatment while others were not. Unfortunately, this information was not available.

#### Echography and hemodynamics measurements

The LV end-diastolic and end-systolic diameters (LVEDD, LVESD), cavity wall thicknesses (interventricular septum (IVS) and LV posterior wall (LVPW)), and LVEF were measured by standard transthoracic echocardiogram according to the American Society of Echocardiography guidelines using commercially available ultrasound systems with phased-array transducers Sonos 5500 or iE33 (Philips Medical Systems, Best, the Netherlands).

#### Collagen

Endomyocardial biopsy samples were taken as part of the clinical treatment for a selection of HF patients (HF_C_: *n* = 8, HF_0_: *n* = 8) to measure collagen content. Samples were dyed with Sirius red, and the CVF was calculated as the fraction of dyed regions in the thin tissue slices. Biopsies were extracted with no specific orientation and therefore provided no directional information with respect to the muscle fibers.

We sought to relate changes in CVF to changes in passive mechanical stiffness by performing a literature survey of biopsy-based comparisons of patients with and without HF. The latter patient category yielded control CVF levels, assumed to resemble healthy heart tissue.

### Finite-element model and simulation

#### Geometrical configuration

**Figure 1:**
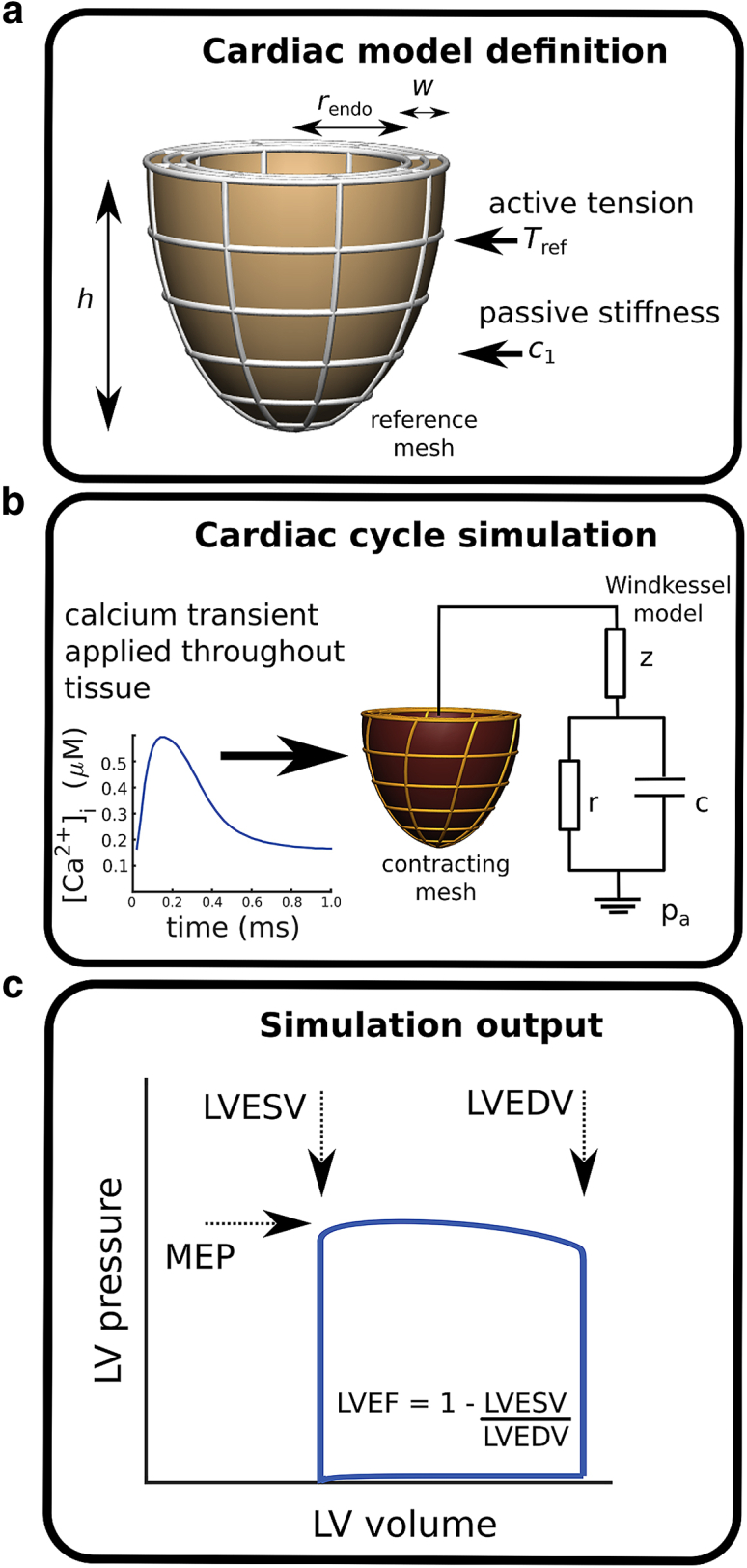
General model structure and simulation workflow. (*a*) The reference unloaded LV is represented as a semiellipsoidal mesh with radius *r*_endo_. Mechanical properties are characterized by a maximal tension *T*_ref_ and a passive stiffness scaling factor *c*_1_ (68). (*b*) The mechanical deformation of the system upon application of a calcium transient (25) is calculated numerically over the time course of several full cycles until reaching a steady state (see the text for the Simulation Protocol) (22). The “Windkessel model” represents the external circulation, parameterized by an aortic-valve resistance *z* = 4.7 kPa ⋅ s ⋅ 1^−1^, arterial compliance *c* = 0.02051/kPa, and peripheral arterial resistance *r* = 113.7 kPa ⋅ s ⋅ 1^−1^ (70). The aortic pressure before ejection is *p*_a_. (*c*) The calculated steady-state phenotypes are the LV end-diastolic and end-systolic volumes LVEDV and LVESV and the maximal ejection pressure MEP. To see this figure in color, go online.

The unloaded LV was constructed as a finite-element mesh of concentric semiellipsoids with eight elements in the azimuthal direction, six in the apex-base direction, and two across the wall (Fig. 1 *a*). The wall thickness was fixed at 10 mm, and the innermost (endocardial) semiellipsoid was 60 mm long. Meshes with different endocardial radii (nine values in the range 20–33 mm) were constructed to simulate LV dilation. Muscle-fiber orientations within the tissue were defined by imposing a rule-based configuration, with elevation angles varying linearly across the wall, from −60° (epicardial surface) to +80° (endocardial) (21, 22).

#### Electromechanical coupling

LV contraction in the real heart is initiated by periodically releasing intracellular calcium to generate tension. This cardiac cycle was simulated within the computational framework described by Land et al. (23, 24), using a reference LV mesh to represent the unloaded LV.

The simulation is driven by a periodic (1 Hz) intracellular calcium stimulus, derived from human-cell measurements (25), applied simultaneously throughout the tissue (Fig. 1 *b*). Tissue contraction is modeled using a set of ordinary differential equations describing the activation of force generation by myosin cross-bridges and actin filaments. Maximal tension is characterized by the tension-scaling parameter *T*_ref_. The phenomenological representation of the physiological behavior includes length and velocity dependences observed in muscle (22, 26, 27). The mechanical response is determined by modeling the tissue as an elastic material that is transverse-isotropic with respect to the muscle fibers and with a stiffness characterized by the scaling parameter *c*_1_ (see Appendix A). The computed LV deformation balances the locally generated tension, the viscoelastic response, and external boundary conditions. The self-consistent solution of the mechanics accounts for the transmission of the generated tension throughout the tissue (including in directions perpendicular to the fibers) as determined by the strain-energy functional and the imposed incompressibility condition.

#### Computational method and boundary conditions

Partial differential equations describing the mechanical force balance were solved subject to tissue incompressibility (28). Spatial boundary conditions were defined by constraining the LV basal plane to lie perpendicular to the apex-base axis, with one mesh node on the interior LV wall fixed in all directions. Another node, located diametrically opposite on the LV interior, was constrained to move along the axis connecting the fixed node to prohibit rigid-body rotation. Pressure boundary conditions were defined by specifying the aortic pressure *p*_a_, which was coupled to the LV via a three-element Windkessel model (Fig. 1 *b*).

#### Simulation protocol

The cardiac cycle was simulated as previously described, implementing the basic steps of the cardiac cycle (23, 24). In summary, a phenomenological model of diastolic filling governs LV inflation toward a set end-diastolic pressure of 0.5 kPa (4 mmHg). Upon stimulating the muscle with a measured calcium transient (25), the LV volume becomes fixed to simulate the closing of the mitral valve and the subsequent isovolumic contraction. Active tension is thus generated within the tissue along the fiber directions (and transmitted throughout the tissue mass as determined by the tissue mechanical properties and the incompressibility constraint), resulting in a sharp LV pressure rise. Upon reaching the preset aortic pressure *p*_a_, the isovolumic constraint is removed, representing the opening of the aortic valve, and the Windkessel model governs blood ejection from the LV until the volume-flow direction is reversed (closing of the aortic valve). Fixing the LV volume again in this state, isovolumetric relaxation is initiated, terminating when the LV pressure attains a preset diastolic pressure (0.2 kPa = 1.5 mmHg). Diastolic filling is then reactivated as the mitral valve reopens. The process is repeated until the model converges to a steady-state limit cycle, typically after three contractions (when the LV end-diastolic volume (LVEDV) varies by no more than 0.5% between successive cycles). The mesh-node coordinates and LV volume and pressure are recorded as functions of time over the limit cycle. A representative pressure-volume loop is plotted in Fig. 1 *c*.

### Combining simulations and measurements

#### Parameter space

The simulation setup was guided by the clinical results summarized in Table 1 to emulate the observed phenotypes. In summary, the principal phenotypes differentiating the three cohorts are LVEF, LVEDD, and LVESD. Properties that determine LVEDD include the LV geometry, the passive mechanical stiffness of the tissue, and the diastolic filling pressure. In the absence of experimental data characterizing diastolic filling, we assumed a constant end-diastolic pressure *p*_ed_ for all simulations and amalgamated it with the mechanical stiffness *c*_1_ into a phenomenological scaling parameter: *c*_1_ ∼ *c*_1_/*p*_ed_. The systolic properties represented by LVEF and LVESD additionally require a consideration of the active tension magnitude, scaled phenomenologically by the model parameter *T*_ref_. Because MEP was the same in all cohorts, the aortic pressure *p*_a_ was assumed constant. We therefore defined the model parameter space as comprising the reference (unstrained) LV endocardial diameter 2 × *r*_endo_ and treating *T*_ref_ and *c*_1_ as phenomenological parameters that cumulate the entire ensemble of cellular-level mechanisms defining active and passive mechanics.

**Table 1:** Clinical Phenotypes Measured for each of the Patient Cohorts

Clinical phenotype	Healthy (*N* = 10)	HF_C_ (*N* = 22)	*p*(HA → HF_C_)	HF_0_ (*N* = 25)	*p*(HA → HF_0_)	*p* (HF_C_ → HF_0_)	*p*_grad_ (HF_C_ → HF_0_)
LVEDD (mm)	50 ± 6	55 ± 7	0.06	57 ± 7	0.01	0.4	<10^−5^
LVESD (mm)	35 ± 6	46 ± 8	0.003	47 ± 10	0.001	0.8	<10^−5^
IVS (mm)	9.4 ± 1.4	8.4 ± 1.6	0.10	8.6 ± 1.4	0.12	0.6	0.4
LVPW (mm)	9.3 ± 1.3	8.6 ± 1.2	0.16	8.6 ± 1.2	0.12	1.0	1.0
MEP (mmHg)	140 ± 12	134 ± 18	0.35	141 ± 22	0.9	0.3	1.0
MinAP (mmHg)	81 ± 8	85 ± 14	0.37	81 ± 12	0.84	0.5	0.0004
LVEF (%)	58 ± 3	35 ± 13	<10^−5^	34 ± 14	<10^−5^	0.6	ND
Heart rate (bpm)	68 ± 15	78 ± 18 (*n* = 8)	0.16	66 ± 11 (*n* = 8)	0.8	0.05	1.0
CVF (%)	ND	12 ± 10	ND	8.4 ± 5.6	ND	0.3	ND

Values indicate the means and SDs, measured for each of the patient cohorts, as illustrated in the histograms in Fig. S1: healthy hearts (*N* = 10), HF_C_ (*N* = 22), and HF_0_ (*N* = 25). The quoted *p* values evaluate the discrepancy between each HF cohort (HF_C_, HF_0_) and the healthy hearts. Collagen fractions were measured in biopsies taken from patients in the HF_C_ (*n* = 6) and HF_0_ (*n* = 8) groups. No healthy-patient biopsies were available within this study. LVEDD, LV end-diastolic diameter; LVESD, LV end-systolic diameter; IVS, interventricular septum thickness; LVPW, LV posterior wall thickness; MEP, maximum ejection pressure; MinAP, minimum aortic pressure; LVEF, LV ejection fraction; HR, heart rate; CVF, collagen volume fraction; ND, no data.

We constructed a mesh of fixed basal radius *r*_endo_ = 22.3 mm to represent cohort HA but allowed *r*_endo_ to vary to accommodate LV dilation in HF. MEP and minimal aortic pressure (MinAP) values are similarly homogeneous across cohorts, and the aortic pressure was therefore set to a constant *p*_a_ = 15 kPa (112 mmHg). A constant wall thickness *w* = 10 mm, consistent with the echo measurements, was also adopted in all the simulations.

The strength of active tension was varied by tuning the tension-scaling parameter *T*_ref_ (corresponding to maximal active tension, when all myosin cross-bridges are bound to actin) around 120 kPa (as measured in isolated myofibrils (29)). Passive stiffness was varied by tuning the stiffness-scaling parameter *c*_1_ (Eq. 4) to match the simulated to the observed LVEDD. The simulations were repeated to map the parameter space defined by

(1) $$\{\begin{array}{cc} c_{1} & =0.6\;\ldots \;8.0\;\text{kPa}\\ T_{\text{ref}} & =80\;\ldots \;180\;\text{kPa}\\ r_{\text{endo}} & \;\;\;\;\;\;=20.0\;\ldots \;33.0\;\text{mm} \end{array}$$

To confirm that these conditions satisfactorily covered the range of plausible physiological scenarios, we checked that no significant domains of this space were truncated by the choice of parameter bounds, as explained in the Supporting Materials and Methods.

#### Comparing simulations and measurements

Each simulation outputs the time courses for the LV pressure, volume, and node coordinates over a full steady-state cycle, thereby allowing a mapping of the simulation parameters to the phenotype measurements:

(2) $$\left\{c_{1},T_{\text{ref}},r_{\text{endo}}\right\}\to \left\{\text{LVEDD},\text{MEP},\text{LVEF}\right\}$$

Neither cohort can be represented by a unique parameter set, firstly because of the phenotype variability and secondly owing to the “many-to-one” nature of the mapping (Eq. 2). Our analysis, therefore, aimed to determine the distribution of parameter transformations that plausibly describe the development of HF as follows.

**Figure 2:**
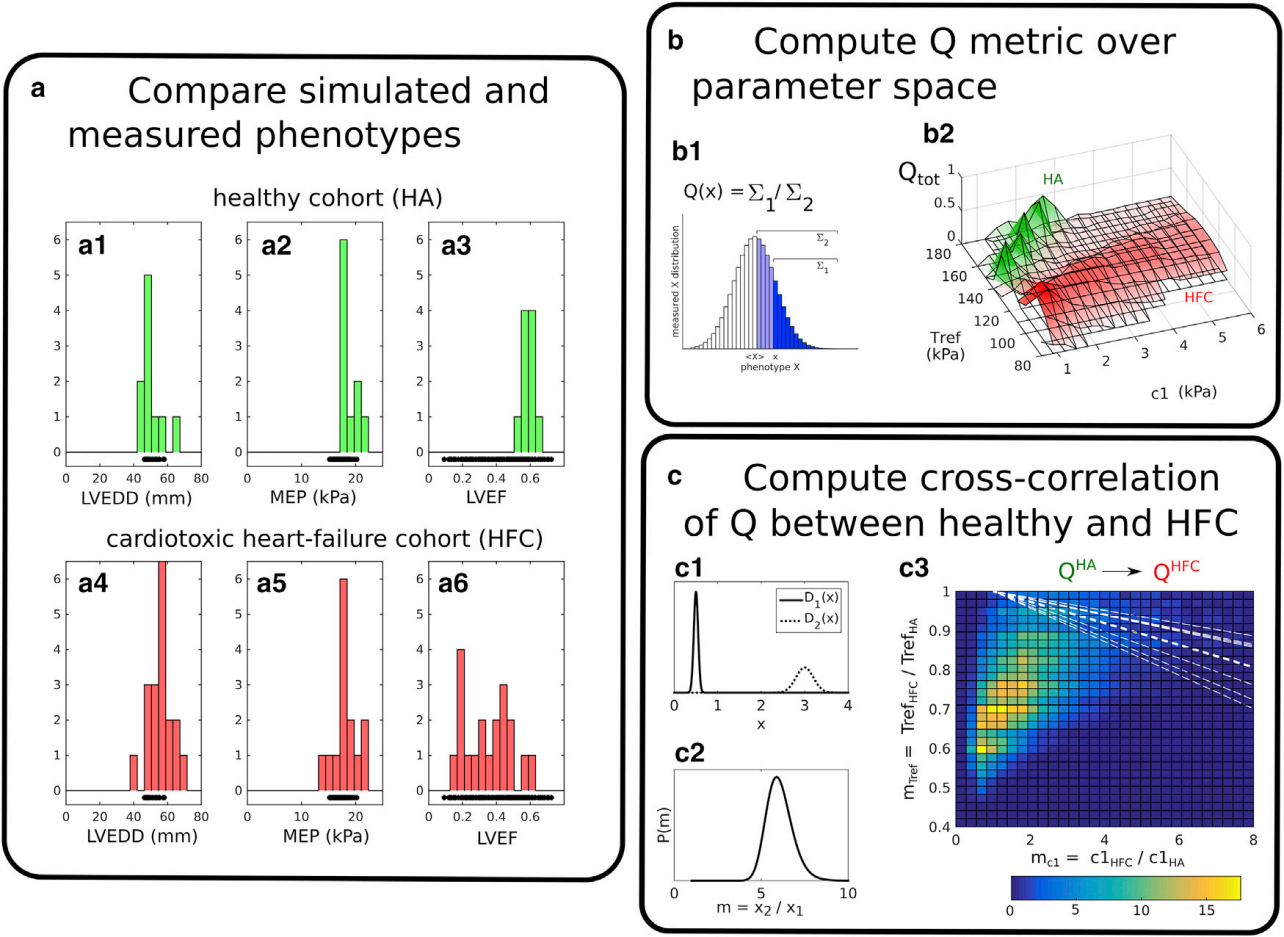
Analysis workflow for mapping phenotypes differences to model parameter transformations. Simulated results are compared to the measured phenotype distributions (LVEDD, MEP, and LVEF, taken from Fig. S1) for the healthy (*a1*–*a3*) and HF_C_ cohorts (*a4*–*a6*). The black marks below the histograms indicate the phenotypes predicted by the simulations (assuming constant *r*_endo_ = 22.3 mm and aortic pressure *p*_a_ = 15 kPa = 112 mmHg). (*b*) The consistency of individual simulations with the measured phenotype distributions was assessed through the metric *Q*. The principle for computing *Q* for a generic phenotype distribution *X* is illustrated in (*b1*). *Q* = Σ_1_/Σ_2_, with Σ_1_ and Σ_2_ representing integrals over the distribution, bounded by *x* and the distribution mean 〈X〉. (*b2*) Maps of *Q*_tot_ = *Q*_LVEDD_ × *Q*_ME_ × *Q*_LVEF_ are then computed over the parameter space (Eq. 1) separately for each cohort. The distribution *P* of parameter fold changes is computed for the pair of *Q*_tot_ functions, as described in the Appendix B. A schematic illustration for two one-dimensional functions *D*_1_(*x*) and *D*_2_(*x*) is shown in (*c1*), with the corresponding *P*(*m*) plotted in (*c2*). The two-dimensional $P\left(m_{{\text{c}}_{1}},m_{{\text{T}}_{\text{ref}}},m_{{\text{r}}_{\text{endo}}}=1\right)$ obtained from (*b2*) is mapped out in (*c3*). The white dashed lines indicate the region where the decrease in *T*_ref_ may be accounted for by an increase in the CVF, as calculated in Appendix C. To see this figure in color, go online.

The analysis workflow is illustrated in Fig. 2 for the transition between HA and HF_C_. Fig. 2 *a* compares the measured phenotypes, represented by histograms, with a slice through the simulated data set corresponding to constant *r*_endo_ = 22.3 mm, represented by black points plotted below the histograms. Each simulation yields phenotype values that either conform with one or both patient cohorts (when the values lie within the histogram ranges) or with neither (when the value lies outside the measurement bounds).

We define a metric *Q* ∈ [0, 1] to quantify the degree of agreement between a simulated and a measured phenotype for a given cohort, taking into account the variability of that phenotype within the cohort, as illustrated in Fig. 2 *b1*. For a given value of *x* of a generic phenotype, we define $Q\left(x\right)\equiv {\text{Σ}}_{1}/{\text{Σ}}_{2}$, where Σ_1_ is the number of measurements that are more distant than *x* from the distribution mean 〈x〉 and Σ_2_ is the total number of measurements that are on the same side of 〈x〉 as *x*, as indicated. Thus, *Q* = 1 corresponds to a simulation for which *x* coincides with 〈x〉 (representing maximal consistency), whereas *Q* = 0 signifies that the simulation lies outside the measurement range (no consistency). An overall consistency metric *Q*_tot_ ∈ [0, 1], covering all the phenotypes, is then computed by multiplying the particular *Q* values:

(3) $$Q_{\text{tot}}\equiv Q_{\text{LVEDD}}\times Q_{\text{MEP}}\times Q_{\text{LVEF}}$$

For illustration, the surface plots in Fig. 2 *b2* map the functions *Q*_tot_ obtained separately for HA (*green*) and HF_C_ (*red*), assuming *r*_endo_ = 22.3 mm for both cohorts (i.e., the slice through the domain of Eq. 1 corresponding to no LV dilation). The peaks in the landscape identify the regions of the parameter space that are more strongly associated with one or the other cohort.

#### Estimating parameter transitions from healthy to failing hearts

Using the *Q*_tot_ distributions, we determined what parameter transformations, when applied to the healthy heart data set, could most plausibly represent the development of HF. A “transformation,” in this context, is defined as the ratio $m_{x}\equiv x_{2}/x_{1}$ between values of parameter *x* in cohorts 1 and 2. To quantify the distance from cohort 1 to cohort 2, we sample all possible pairs of points in the parameter space (Eq. 1), calculate the corresponding $m_{c_{1}}$, $m_{{\text{T}}_{\text{ref}}}$, and $m_{{\text{r}}_{\text{endo}}}$ ratios, and construct the distribution $P_{1\to 2}\left(m_{c_{1}},m_{{\text{T}}_{\text{ref}}},m_{{\text{r}}_{\text{endo}}}\right)$ of these ratios, weighted by the associated *Q*_tot_ values for cohorts 1 and 2. The calculation, defined formally in the Appendix B, is akin to calculating the cross correlation between the two *Q*_tot_ distributions. Fig. 2 *c* illustrates a representative two-dimensional slice through this function, corresponding to the special case of no LV dilation, i.e., $P_{\text{HA}\to \text{HFC}}\left(m_{c_{1}},m_{{\text{T}}_{\text{ref}}},m_{{\text{r}}_{\text{endo}}}=1\right)$. We interpret the magnitude of *P* as measuring the relative ability of a given transition to link the healthy to the diseased cohort.

## Results

### Clinical phenotypes

Phenotypes are compared in Table 1 (see *histograms* in Fig. S1). The *p*-values associated with each mean were calculated using two-sample *t*-tests to assess the discrepancies between the healthy and each diseased cohort. Both HF_C_ and HF_0_ differ mostly markedly from HA in terms of LVEF (a 40% decrease on average, *p* < 10^−5^), reflecting the aptitude of LVEF as an indicator of HF. This effect is accompanied by a significant increase in LVESD (*p* ≤ 0.003) for both HF cohorts, also consistent with impaired ejection. In contrast, MEP, MinAP, IVS, and LVPW differ much less significantly between the cohorts (*p* > 0.1). However, LVEDD is increased in HF by a factor of order 10%, albeit less significantly for HF_C_ (*p* = 0.06) than for HF_0_ (*p* = 0.01). None of the phenotypes, including LVEF, LVEDD, and LVESD, show any significant difference when comparing HF_C_ with HF_0_ directly (*p*_(HFC → HF0)_ > 0.3).

**Figure 3:**
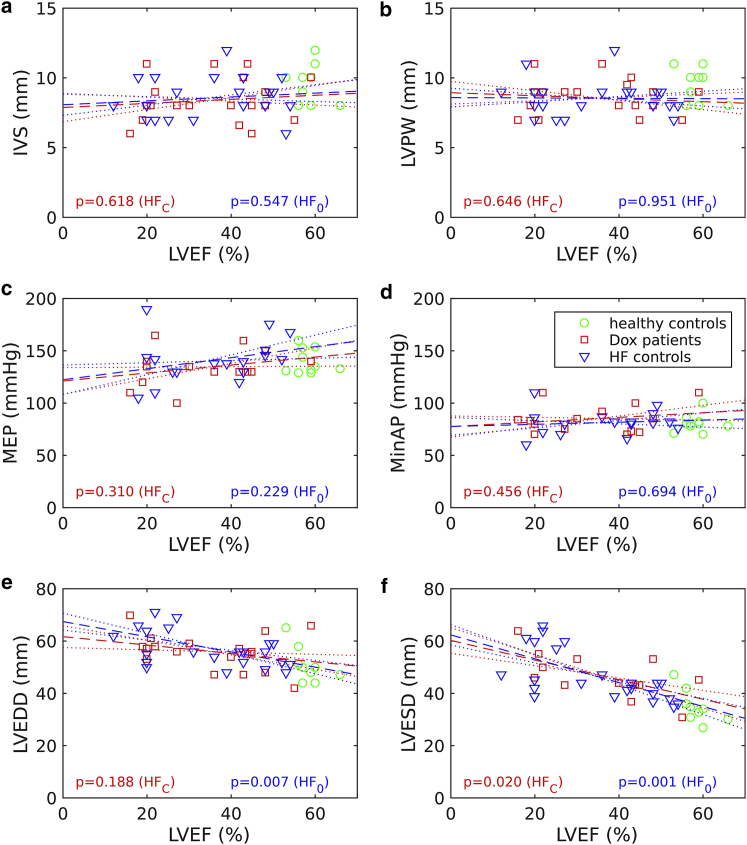
Dependences of echocardiographic and pressure phenotypes on the LVEF across the patient cohorts: (*a*) interventricular septum thickness, (*b*) LVPW thickness, (*c*) MEP, (*d*) minimal aortic pressure, (*e*) LVEDD, (*f*) LVESD. The black dashed lines represent the linear fit through all the data points, and the dotted lines represent one SD in the calculated slope above and below the line of best fit. The *p*-values assess the deviation of the fitted slope from zero. To see this figure in color, go online.

The above analysis, based purely on the individual phenotype distributions, remains inconclusive with regard to distinguishing between HF_C_ and HF_0_ in terms of LVESD and LVEDD. Fig. 3 compares the linear regression coefficients for each phenotype with respect to LVEF, with significance (*p*_grad_) assessed in the last column of Table 1 (30). No significant difference is again observed for MinAP, MEP, IVS, or LVPW (*p*_grad_ > 0.4). However, LVEDD, LVESD, and MinAP all display significant differences (*p* < 0.0005), which supports the exploration of the simulation parameter space defined by *c*_1_, *T*_ref_, and *r*_endo_ (Eq. 1).

### Collagen measurements

**Figure 4:**
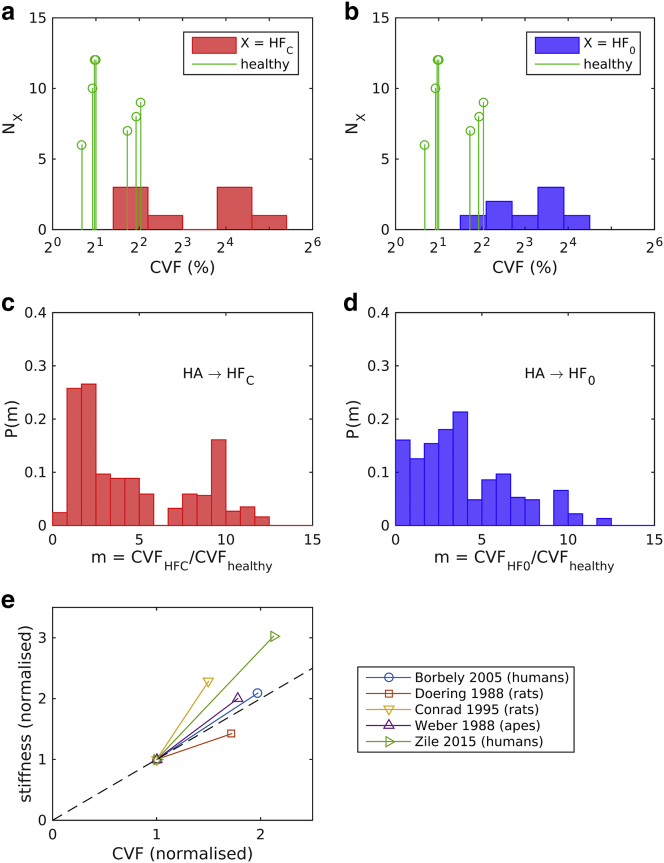
Histograms of the CVF distribution are shown for the (*a*) HF_C_ (*red*, mean 10.3 ± 6.7%) and (*b*) HF_0_ patient cohorts (*blue*, mean 8.4 ± 5.6%). The green data points in (*a*) and (*b*) represent the CVF measurements done on healthy hearts, as reported in the literature (31, 32, 33, 34, 35, 36, 37) (mean 2.6%, weighted according to the number of measurements in each publication). The histograms in (*c*) and (*d*) represent the distributions of the CVF ratios computed from (*a*) and (*b*), respectively. (*e*) A literature survey of the relation between passive tissue stiffness and collagen volume fraction (CVF) is given, comparing healthy hearts and cases of diastolic HF in different species (34, 43, 64, 65, 66). The CVF and stiffness values are normalized by their respective values corresponding to a healthy heart to highlight the relative change in CVF and stiffness values. The dashed line has unit gradient. To see this figure in color, go online.

CVF was measured in endocardial biopsies taken from a subset of HF_C_ and HF_0_ patients (Fig. 4, *a* and *b*; Table 1). Healthy CVF values were estimated from a literature survey of biopsy measurements performed on patients testing negative for cardiovascular disease (*green data* in Fig. 4, a and b, in which the height of each data point represents the number of measurements performed in the corresponding study (31, 32, 33, 34, 35, 36, 37)). The mean CVF values for the HF_C_ (10.3%) and HF_0_ (8.4%) cohorts differ by factors of 4.0 and 3.2, respectively, relative to the mean healthy CVF (2.6%).

Variations in cardiac collagen content have been shown to correlate with passive mechanical stiffness (38, 39, 40, 41). Although other factors are important (e.g., collagen type and the degree of cross-linking (42)), tissue stiffness is consistently reported to increase with CVF, both in isolated cardiomyocytes (34) and in the intact myocardium (43). An approximately proportional relationship is apparent in Fig. 4 *e*.

### Simulations

**Figure 5:**
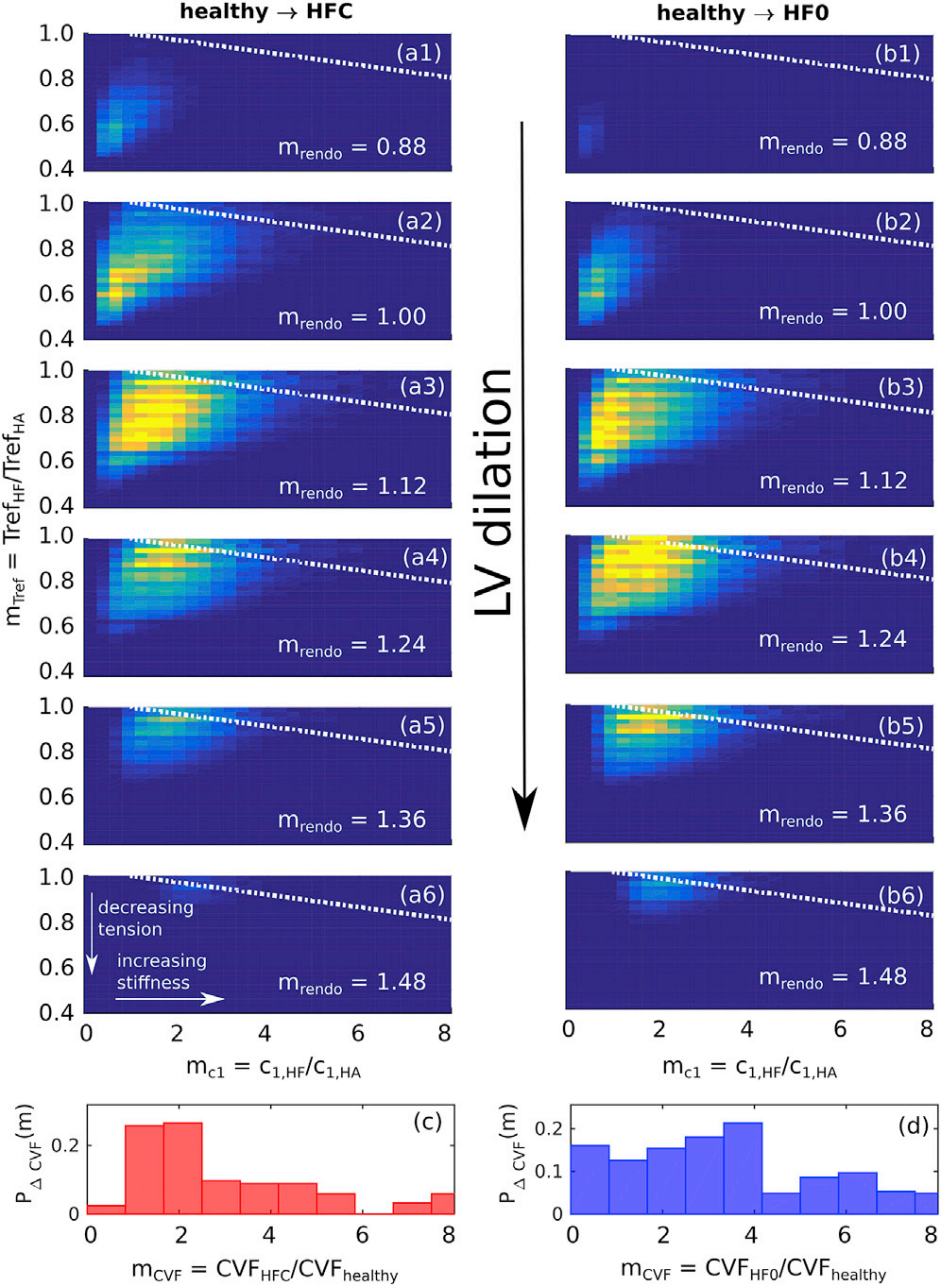
Transition maps connecting *(a*1–*a*6) healthy to HF_C_ hearts and (*b*1–*b*6) healthy to HF_0_ hearts by varying *c*_1_ (*horizontal axis*), *T*_ref_ (*vertical axis*), and *r*_endo_ (successive images) by factors $m_{x}\equiv x_{\left(\text{HF}\right)}/x_{\left(\text{HA}\right)}$. The dotted white lines represent the average boundary over which the lowering of *T*_ref_ in the failing hearts may be explained by a reduction of the MVF due to increased CVF (see text). The estimated CVF fold-change distributions *P*_*Δ*CVF_(*m*_CVF_) (see Fig. 4, *c* and *d*), corresponding to the cohort pairs in (*a*) and (*b*), are reproduced in (*c*) and (*d*), respectively. To see this figure in color, go online.

The main results of this work are shown in Fig. 5, which depicts $P_{\text{HA}\to {\text{HF}}_{\text{C}}}$ and $P_{\text{HA}\to {\text{HF}}_{0}}$ as functions of $m_{c_{1}}$, $m_{\text{Tref}}$, and $m_{\text{rendo}}$. The horizontal axes represent increasing *c*_1_, the vertical axes increasing *T*_ref_, and successive images denote LV dilation with increasing *r*_endo_. We interpret each distribution as a measure of the plausibility that a given combination of changes in *c*_1_, *T*_ref_, and *r*_endo_ reproduces the development of the HF phenotypes, starting from the healthy configuration, either in the presence (Fig. 5, *a1–a6*) or absence (Fig. 5, *b1–b6*) of doxorubicin exposure.

The results for transitions to HF_C_ and HF_0_ both display a correlation between $m_{c_{1}}$ and $m_{\text{Tref}}$, i.e., a decrease in *T*_ref_ varies inversely with an increase in *c*_1_, with the balance depending on the extent of LV dilation, as indicated by the shift in the distribution along an upward diagonal with increasing *r*_endo_. We observe a stronger dilation in the case of the HF_0_ transition (mean $\langle m_{{\text{r}}_{\text{endo}}}\rangle =1.21$, averaged over the whole distribution) than for HF_C_ ($\langle m_{{\text{r}}_{\text{endo}}}\rangle =1.13$).

To further constrain the computed distributions to the patient data, we related the biopsy CVF measurements to the stiffness (*horizontal*) axes of Fig. 5, *a* and *b*. We estimated the expected change in *c*_1_ by assuming a proportionality with the change in CVF *m*_CVF_, as suggested by the literature reviewed in Fig. 4 *e*. An effective distribution of *m*_CVF_ was calculated by cross-correlating the healthy and failing-heart CVF distributions shown in Fig. 4 (see Appendix B for details). The resulting distributions *P*_CVF_ (Fig. 4, *c* and *d*) characterize the distributions of ratios *m*_CVF_ that describe the difference in CVF between the cohorts. For the cases of both HF_C_ and HF_0_, *P*_CVF_ covers an order of magnitude, with the bulk of the distribution being in the range *m*_CVF_ < 4, suggestive of a stiffness increase up to threefold and a *T*_ref_ decrease ranging from ∼0 to 20%. The HF_C_ case, however, is more strongly weighted toward lower values (*m*_CVF_ < 2), suggesting a stronger bias toward decreased *T*_ref_ (by ∼10–30%).

### Impact of CVF on contraction

Beside its effect on passive stiffness, an increase in CVF may in principle also cause a reduction in net active tension generation by virtue of the fact that muscle tissue is replaced by noncontractile collagen (cf. replacement fibrosis (44)). The corresponding effective decrease in *T*_ref_ is independent of the ability of cardiac muscle to generate tension.

The results of Fig. 5 indicate that the measured phenotypes can be explained by a combination of changes in the model parameters associated with the passive (*c*_1_) and active properties (*T*_ref_). To assess the extent to which the effective reduction in *T*_ref_ can be accounted for purely as a result of the CVF change, we assumed the simplest scenario, namely an inverse correlation between CVF and the volume fraction of the contracting myocardium, i.e., muscle volume fraction (MVF) = 1 − CVF; and secondly, a linear scaling of the contractile strength with MVF. We hence estimated the decrease in active tension associated with a given increase in *c*_1_ using the formula derived in the Appendix. The dashed lines in Fig. 2 *c3* represent the expected effective decrease in *T*_ref_ arising solely from an increase in collagen (where each *dashed line* corresponds to a different value of healthy heart CVF, serving as a reference value). The regions of the map located above these dashed lines thus represent regimes where the decrease in *T*_ref_ can be explained in terms of reduced MVF rather than through a reduction of muscular contraction. In all the cases considered, this region covered no more than 9% of the total integral. This supports the hypothesis that the extent of decrease in *T*_ref_ required to account for HF cannot be fully explained by a simple decrease in muscle density. Consequently, a greater contribution to the decrease in *T*_ref_ must have other causes, e.g., sarcomere density or alignment.

### Confounding factors

Owing to the natural heterogeneity of patient cohorts and the limited scope of available measurements, our simulations are inevitably approximations of the physiological system. To assess the significance of some potential confounding factors, we performed the following additional simulations.

The recruited patient cohorts, summarized in Tables S1–S3, show the HA controls being predominantly male and the HF_C_ and HF_0_ cohorts being more biased toward females (breast cancer patients). Among various gender dependences reported in the cardiac system (45, 46, 47, 48), one particular difference is in the LV mass and LVEDV, which are on average 70% of the values in males (49). To test the potential impact of this discrepancy on our conclusions, we repeated simulations after scaling the LV meshes to 89% of their value (0.89 = 0.70^1/3^) in all dimensions to represent “female” meshes. Simulations were repeated and analyzed as previously, using these smaller meshes for mapping the HF_C_ and HF_0_ data. The results (Fig. S3) show no qualitative difference with those in Fig. 5.

The clinical measurements associate HF with a dilation of the LV cavity. The long-axis dimension of the LV cavity was not measured, and the simulations therefore assumed a constant long-axis length of 60 mm. To estimate the potential impact of LV elongation, we repeated the simulations and analysis after scaling the long axis in proportion to *r*_endo_. The results show no qualitative difference with our findings (Fig. S4).

The LV filling pressure was assumed to be fixed for all the simulations but is known to increase in HF (50, 51). This property was, however, not characterized in our patient cohorts. To estimate the potential sensitivity of our conclusions to this effect, we repeated the simulations by doubling the filling pressure. As expected, the results display an increase in the stiffness ratio $m_{\text{c}1}$ (Fig. S5), but the general qualitative comparison between the HF_C_ and HF_0_ results remains unchanged, with the former showing a stronger contribution from decreasing *T*_ref_.

Some authors have reported a modification of the fiber-orientation distribution in cases of dilated cardiomyopathy and after pressure overload, with fibers becoming more oblique relative to the LV wall cross section (52, 53). To assess the potential impact of changes in fiber orientation on our conclusions, we repeated simulations using the “dilated” mesh with *r*_endo_ = 30 mm, making the endocardial and epicardial fiber directions either more oblique (+10°) or more circumferential (−10°) relative to the baseline fiber configuration. The effect on the simulated phenotypes was generally minimal. In particular, the maximal change in LVEF was ∼3% (Table S6), significantly less than the >20% caused by HF. This effect arguably does not affect our main qualitative conclusions.

## Discussion

This study aimed to employ for the first time, to our knowledge, a biomechanical model to provide a framework for interpreting clinical data specifically in the context of doxorubicin cardiotoxicity. By mapping computational simulations of the cardiac cycle onto clinically accessible functional phenotypes, we evaluated the relative contributions of changes in the passive, active, and morphological LV properties to the onset of the mechanical phenotypes characterizing doxorubicin-induced and conventional HF. This in turn provided a means for identifying patterns characteristic of doxorubicin-induced HF specifically.

We found no difference in blood pressure in HF patients who had or had not undergone doxorubicin chemotherapy. On the other hand, differences in the correlations between active, passive, and anatomical properties were detected by quantifying the degree of consistency between simulated and measured phenotypes. The results, summarized in Fig. 5, suggest that depending on the extent of LV dilation, HF cardiac phenotypes can be explained by a combination of increased stiffness and decreased strength of muscle contraction. In the case of patients with doxorubicin-induced HF, the clinical data are more consistent with 1) less LV dilation and 2) a predominance of reduced active tension over increased passive stiffness compared to cases of HF unrelated to chemotherapy.

The clinical effects of anthracyclines on cardiac function are well known and documented (12, 54, 55, 56, 57, 58). However, this information has yet to turn into tailored or improved diagnosis and treatment. Experimentally, many in vivo and ex vivo studies have been performed to examine immediate or short-term drug impact (20, 56, 59, 60). However, translating these findings to the clinical case that can take years to manifest is challenging. The long-term nature of doxorubicin cardiotoxicity also makes the systematic tracking—or retrospective reconstruction—of its development problematic. This study, therefore, sought to detect first-order effects by linking measurable pathophysiological cardiac mechanical phenotypes to classes of mechanistic properties of the heart and to yield insight into the dominant underlying mechanisms.

Despite the simplifications inherent in the analysis, the results open potential perspectives for refining the diagnosis and treatment options available to cardiotoxic HF patients that are absent in current clinical practice. At present, treatment for cardiotoxicity is instigated only once HF has materialized. An improved awareness of the dominant contributing factors leading to HF might facilitate the early detection of those emerging factors before the onset of HF. Treatment options may also be improved at later stages of HF. As stated earlier, cardiotoxic HF patients currently receive the same standard treatment for HF that focuses on reversing external symptoms rather than targeting underlying causes (e.g., in cases of reduced LVEF, enhancing LVEF through a reduction of arterial tension by *β* blockers and ACE inhibitors) (7). Thus, a future treatment of cardiotoxic HF may emphasize, through appropriate medication, the restoration of contractile function. The choice of treatment may be further guided by a closer monitoring of the patients after the initiation of chemotherapy. For example, an HF patient showing evidence of significant LV dilation over the course of chemotherapy might potentially benefit from a treatment targeting passive mechanical properties. The drug pirfenidone inhibits transforming growth factor *β*, which plays a central role in activating cardiac fibrosis and has been shown in animal studies to reduce passive diastolic stiffness while not restoring cardiac contractility (61).

By identifying classes of mechanisms that most likely account for HF phenotypes and by accommodating for the natural variety within cohorts, our approach sought to discern trends that would escape notice in experiments focusing on individual cases or specific mechanisms. Ultimately, this may provide a framework for contextualizing known results and guiding more focused and detailed clinical investigation. Within this perspective, our results suggest a greater emphasis on the deterioration of active contractile function following from doxorubicin chemotherapy than in more general cases of HF.

### Limitations

Like all patient modeling studies, this study has limitations. HF is a heterogeneous disease, and we recruited limited patient numbers. Chemotherapy patients typically receive a range of simultaneous medications rather than being limited to doxorubicin (see Table S1). In addition, most of the patients referred to the cardiac clinic were already receiving HF medication (Tables S4 and S5). Hence, we were limited by the available clinical data, so we are not powered to detect negative findings. Within this constraint and given the role of subjectivity in diagnosing HF, we aimed to minimize bias by limiting the cohorts to patients recruited at the same hospital.

Several authors have reported that a portion of the generated stress is exerted transversely relative to the mean local fiber orientation (62, 63). This study sought to identify first-order effects on cardiac behavior, assuming that the generation of active stress occurs exclusively in the direction of the muscle fibers (Eq. 5). The potential quantitative impact of transverse stresses on our conclusions remains to be explored.

A further limitation of the approach is its inability to assign functional changes to specific molecular mechanisms, which are instead encapsulated into phenomenological representations of broad classes of phenomena. For example, passive stiffness, quantified by *c*_1_, makes no explicit reference to collagen or titin, known determinants of cardiac tissue stiffness (34, 41, 43, 64, 65, 66). Similarly, a reduction in *T*_ref_ can in principle result from a range of different possible perturbations at the sarcomere level (59, 60, 67). We hope that our findings may ultimately guide future studies.

## Conclusion

We have created the first biomechanical models, to our knowledge, to study long-term doxorubicin-induced cardiotoxicity. This provides a physiologically and physically constrained framework for integrating and interpreting clinical data in terms of underlying mechanisms. We have used these models to provide bounds on the anatomical, tissue, and cellular mechanisms that explain the organ-scale clinical phenotypes within this challenging patient group.

## Author Contributions

A.L. performed the simulations and analysis and wrote the article. S.L. wrote the code for the computational model. J.M., A.R., S.H., and P.S. provided the clinical data. J.K. directed the HeCaToS project that comprised this study. S.A.N. supervised the study and wrote the article.

## APPENDIX A: TISSUE MECHANICS

The passive mechanical behavior was governed via the transverse-isotropic strain-energy functional (68)

(4) $$W=\frac{1}{2}c_{1}\text{exp}\left[c_{2}E_{11}^{2}+c_{3}\left(E_{22}^{2}+E_{33}^{2}+2E_{23}^{2}\right)+2c_{4}(E_{12}^{2}+E_{13}^{2}\right],$$

where ***E*** is the Lagrangian strain tensor expressed relative to the muscle fiber and sheet directions and the parameters *c*_2_ = 8, *c*_3_ = 2, and *c*_4_ = 4 characterized the tissue anisotropy (69). Treating the prefactor *c*_1_ as a phenomenological scaling factor for the overall passive tissue stiffness, we allowed it to vary to preserve the ratios between the different stiffness components. The total local stress tensor is hence defined as

(5) $$T=\partial W/\partial E+T_{\text{a}}e_{1}e_{1}^{\text{T}},$$

where ***e***_1_ is the unit vector along the fiber direction. The self-consistent solution of the mechanics problem in the simulations accounts for the transmission of the generated tension throughout the tissue.

## APPENDIX B: DISTANCE BETWEEN DISTRIBUTIONS

Consider two functions *D*_1_ and *D*_2_ representing the distributions for some metric (e.g., *Q*) for two cohorts 1 and 2. We characterize their distance by considering, in turn, every possible pair of samples (*x*_1_, *x*_2_) taken from *D*_1_ and *D*_2_ and constructing the distribution of the fold change *m* = *x*_2_/*x*_1_ within each pair in going from *x*_1_ to *x*_2_. Formally, for continuous one-dimensional functions *D*_1_(*x*) and *D*_2_(*x*), this fold-change distribution is expressed as

$$P_{1\to 2}\left(m\right)\Delta m=\int_{x}\int_{x'=mx}^{\left(m+\text{Δ}m\right)x}D_{1}\left(x\right)D_{2}\left(x'\right)\;\text{d}x\;\text{d}x'$$

By letting *Δm* → 0, we obtain

(6) $$P_{1\to 2}\left(m\right)=\int D_{1}\left(x\right)D_{2}\left(mx\right)x\;\text{d}x$$

This calculation is illustrated for a generic one-dimensional case in Fig. 2, *c1* and *c2*, where $P_{D_{1},D_{2}}\left(m\right)$ is calculated for two generic one-dimensional distributions *D*_1_(*x*) and *D*_2_(*x*), centered around *x* = 0.5 and *x* = 3, respectively. The peak in $P_{D_{1},D_{2}}\left(m\right)$ represents the average fold change between values sampled in *D*_1_ and *D*_2_ and is centered at *x* = 6. The concept is extended to the three-dimensional parameter space (Eq. 1). A two-dimensional slice $P\left(m_{{\text{c}}_{1}},m_{{\text{T}}_{\text{ref}}},m_{{\text{r}}_{\text{endo}}}=1\right)$ is mapped out in Fig. 2 *c3*, corresponding to the *Q*_tot_ distributions in Fig. 2 *b2*.

## APPENDIX C: ESTIMATING TENSION REDUCTION RESULTING FROM REDUCED MUSCLE FRACTION

The observed increase in CVF between the healthy and failing hearts implies a reduction of the MVF and, arguably, of the net maximal tension that can be generated within the tissue by virtue of the fact that there is less muscle tissue. This would argue for a reduction of the effective *T*_ref_ parameter in the model independently of the contractile strength in the available muscle tissue. We sought to estimate to what extent the reduction in muscle mass may account for the reduction in the effective *T*_ref_. Assuming that *T*_ref_ scales proportionally with MVF = 1 − CVF, we have

$$\frac{T_{\text{ref}}^{\text{HFC}}}{T_{\text{ref}}^{\text{HA}}}=\frac{1-{\text{CVF}}_{\text{HFC}}}{1-{\text{CVF}}_{\text{HA}}}$$

In the light of the observed proportionality between the tissue stiffness and the CVF in Fig. 4 *e*, we may write CVF_HFC_/CVF_HA_ = *c*_1HFC_/*c*_1HA_, giving

(7) $$\frac{T_{\text{ref}}^{\text{HFC}}}{T_{\text{ref}}^{\text{HA}}}=\frac{1-\frac{c_{1\text{HFC}}}{c_{1\text{HA}}}\times {\text{CVF}}_{\text{HA}}}{1-{\text{CVF}}_{\text{HA}}}$$

Equation 7 therefore predicts that even if the contractile strength of muscle tissue remains intact, the effective *T*_ref_ decreases linearly with increasing tissue stiffness *c*_1_, taken as a measure of CVF. The proportionality factor depends on the CVF in healthy hearts (CVF_HA_). Any further decrease in *T*_ref_ is therefore expected to result from a genuine decrease in the contractile strength of muscle tissue rather than simply the amount of muscle tissue available.

## Supporting Material

Document S1. Supporting Materials and Methods, Figs. S1–S5, and Tables S1–S6

Document S2. Article plus Supporting Material
