# Supplementary material for: Balance of Active, Passive, and Anatomical Cardiac Properties in Doxorubicin-Induced Heart Failure
Source: Biophys J. 2019 Jul 29;117(12):2337–48. doi: 10.1016/j.bpj.2019.07.033 (PMC6990149; doi:10.1016/j.bpj.2019.07.033)
Supplement: Document S1. Supporting Materials and Methods, Figs. S1–S5, and Tables S1–S6 [file mmc1.pdf]

Biophysical Journal, Volume *117*

Supplemental Information

Balance of Active, Passive, and Anatomical Cardiac Properties in Doxorubicin-Induced Heart Failure

Alexandre Lewalle, Sander Land, Jort J. Merken, Anne Raafs, Pilar Sepúlveda, Stéphane Heymans, Jos Kleinjans, and Steven A. Niederer

SUPPLEMENTAL INFORMATION
Shifting balance of active, passive, and anatomical cardiac properties in doxorubicin-induced heart failure

A Lewalle, S Land, J Merken, A Raafs, P Sepúlveda,
S Heymans, J Kleinjans, SA Niederer

1 Patient cohorts

All patients were recruited at the Academic Hospital Maastricht, MUMC+ (Maastricht University Medical Center) in a study signed off by the local ethics comitte and adhering to the declaration of Helsinki. Three patient cohorts were considered:

- HF_C (Table S1, $n = 22$): Doxorubicin-treated cancer patients with HF symptoms (e.g., dyspnea, fatigue, edema, reduced LVEF). The HF symptoms developed typically one year or more after the cessation of chemotherapy (average 6 ± 6 years). Chemotherapeutic treatments involved a range of medications and dosage adapted to each patient according to their body mass. Average age = 55 ± 13 years, body mass index 26 ± 3 kg/m^2, gender = 23% male, 77% female.

- HF_0 (Table S2, $n = 25$): Patients suffering from HF (same symptoms as for HF_C) with no history of chemotherapy. Average age = 55 ± 14 years, body mass index = 27 ± 5 kg/m^2, gender (32% male, 68% female).

- HA(Table S3, $n = 10$): healthy adults. Average age = 49 ± 10, BMI = 27 ± 4, gender = 80% male, 20% female.

Table 1. Cardiotoxic heart failure patient cohort (HF_C)

Patient ID	Age	Sex	BMI	Cancer type	Chemotherapeutic agents
68	31	M	24	Non-Hodg lymphoma	Doxorubicin
423	40	F	25	breast cancer	Doxorubicin, epirubicin
510	61	F	20	breast cancer	Doxorubicin, 5-fluorouracil, cyclophosphamide, arimidex (hormonal)
614	65	F	23	breast cancer	Doxorubicin, cyclophosphamide, 5-fluorouracil
695	74	F	23	breast cancer	Doxorubicin, taxotere, cyclophosphamide
715	42	F	28	breast cancer	Doxorubicin, 5-fluorouracil, cyclophosphamide, herceptin
731	59	F	22	breast cancer	Doxorubicin, 5-fluorouracil, cyclophosfamide
796	20	M	23	leukemia	Doxorubicin, idarubicin, cytarabin, cyclophosphamide
1068	49	F	25	breast cancer	Doxorubicin, cyclophosphamide, docetaxel , herceptin
10027	65	F	30	breast cancer	Doxorubicin, epirubicin, tamoxifen
10096	69	M	33	lymphoma	Doxorubicin, cyclophosphamide, rituximab, vincristin, prednison
10130	56	F	30	breast cancer	Doxorubicin, cyclophosphamide, trastuzumab
10198	58	F	31	breast cancer	Doxorubicin, 5-fluorouracil, cyclophosphamide
10201	51	F	27	breast cancer	Doxorubicin, cyclophosphamide, everolimus, exemestane, taxotene
10206	53	M	23	leukemia	Doxorubicin, cyclophosphamide, vincristin, prednison
10217	65	F	23	uterus cancer	Doxorubicin, cyclophosphamide, 5-fluorouracil
10239	62	F	25	breast cancer	Doxorubicine, docetaxel, cyclophosphamide
10247	64	F	26	breast cancer	Doxorubicin, cyclophosphamide, docetaxel, tamoxifen
10252	47	M	27	Hodg lymphoma	Doxorubicin, bleomycine, vinblastin, dacarbazin
10284	65	F	30	breast cancer	Doxorubicin, cyclophosphamide, 5-FU, tamoxifen
10285	61	F	24	breast cancer	Doxorubicin, cyclophosphamide, docetaxel
10312	50	F	27	breast cancer	Doxorubicin, cyclophosphamide, pertuzumab, trastuwumab

Table 2. Non-cardiotoxic heart failure patient cohort (HF_0)

Patient ID	Age	Sex	BMI
144	62	M	27
150	33	M	24
211	22	F	
213	39	M	21
237	71	F	23
245	63	F	25
271	65	F	26
334	43	F	34
346	52	F	28
396	56	F	44
397	22	M	20
407	46	F	27
442	69	F	30
488	60	F	36
523	51	F	26
534	56	F	30
562	47	F	29
617	53	F	38
679	30	M	23
686	63	F	26
724	53	M	22
729	67	M	26
10021	69	F	29
10033	66	M	36
10059	41	F	24

Table 3. Healthy adult cohort (HA)

Patient ID	Age	Sex	BMI
591	43	F	34
596	30	F	19
621	55	M	25
623	65	M	29
629	38	M	22
649	44	M	28
651	45	M	24
708	37	M	29
742	54	M	29
744	54	M	29

Table 4. Heart-failure medication applied to HF_C patients at time of measurements. Numbers in parentheses indicate the dose level (milligram).

Patient ID	Beta blocker	ACE inhibitor	ARB antagonist	Aldosterone antagonist	Diuretics	Digoxine
**– HFC –**						
68	Carvedilol (12.5)		Valsartan (80)	Spironolacton (12.5)	Bumetanide (2)	
423						
510	Bisoprolol (1.25)					
614						
695	Metoprolol (200)				Bumetanide (1)	(0.0625)
715		Captopril (25)			Furosemide (40)	
731					Furosemide (20)	
796		Lisinopril (10)			Furosemide (20)	
68	Carvedilol (12.5)		Valsartan (80)	Spironolacton (12.5)	Bumetanide (2)	
10027						
10096	Carvedilol (25)	Perindopril (2)			Bumetanide (2)	
10130						
10198	Sotalol (160)	Perindopril (5)			Bumetanide (1)	
10201						
10206						
10217	Metoprolol (150)	Perindopril (2)		Spironolacton (12.5)	Bumetanide (4)	(0.625)
10239	Metoprolol (50)	Perindopril (4)		Eplerenon (25)	Furosemide (40)	
10247	Metoprolol (100)		Irbesartan (150)			
10252						
10284	Carvedilol (12.5)	Perindopril (4)		Eplerenon (50)	Bumetanide (1)	
10285	Nebivolol (1.25)	Perindopril (2)		Eplerenon (25)	Bumetanide (2)	
10312						

Table 5. Heart-failure medication applied to HF_0 patients at time of measurements. Numbers in parentheses indicate the dose level received in milligrams.

Patient ID	Beta blocker	ACE inhibitor	ARB antagonist	Aldosterone antagonist	Diuretics	Digoxine
**– HF0 –**						
144	Bisoprolol (2.5)	Captopril (75)			Furosemide (40)	
150	Metoprolol (150)		Valsartan (160)		Furosemide (40)	(0.25)
211	Carvedilol (75)	Lisinopril (10)			Furosemide (20)	
213	Bisoprolol (5)	Quinapril (20)				
237	Carvedilol (25)	Perindopril (2)			Furosemide (40)	
245	Bisoprolol (7.5)	Ramipril (2.5)			Bumetanide (0.5)	
271	Metoprolol (50)	Fosinopril (5)			Furosemide (40)	
334	Carvedilol (25)	Enalapril (20)		Spironolacton (25)	Bumetanide (2)	
346	Sotalol (160)	Perindopril (10)				
396	Carvedilol (6.25)		Valsartan (80)		Bumetanide (2)	(0.25)
397						
407		Quinapril (10)			Furosemide (80)	
442						
488					Hydrochloorthiazide (12.5)	
523	Metoprolol (100)	Quinapril (40)			Hydrochloorthiazide (12.5)	
534	Metoprolol (50)	Ramipril (10)				
562	Bisoprolol (2.5)	Perindopril (4)			Bumetanide (1)	
617	Bisoprolol (2.5)					
679	Bisoprolol (2.5)	Perindopril (2)				
686	Carvedilol (6.25)	Perindopril (2)				
724	Nebivolol (5)	Perindopril (4)		Eplerenon (25)	Bumetanide (2)	
729						
10021	Nebivolol (2.5)				Furosemide (80)	
10033	Carvedilol (12.5)	Perindopril (8)		Eplerenon (25)		
10059	Carvedilol (50)	Lisinopril (20)		Spironolacton (25)	Hydrochloorthiazide (12.5)	

2 Results

2.1 Clinical phenotypes

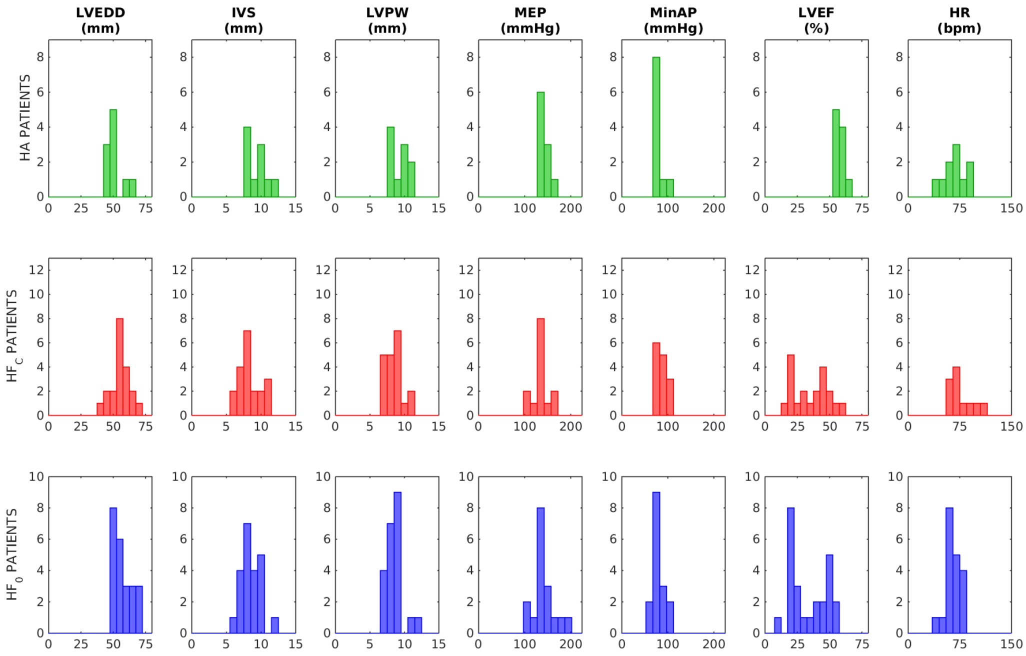

Figure 1. Cardiac phenotypes measured for three patient cohorts: healthy adults (HA), patients with HF following cardiotoxic chemotherapy (HF_C), and HF patients who have not undergone chemotherapy (HF_0). The phenotypes are the LV end-diastolic diameter (LVEDD), intraventricular septum (IVS), LV posterior-wall thickness (LVPW), maximum ejection pressure (MEP), minimum aortic pressure (MinAP), LV ejection fraction (LVEF), and heart rate (HR). Mean values, standard deviations, and p values are listed in Table 1.

2.2 Parameter bounds

The explored range of simulation parameters (Eq. 1) seeks to comprehensively span the physiological scenarios that plausibly give rise to the measured phenotypes. Equivalently, this requires that the distribution of metric $Q_{\mathrm{tot}}(c_1, T_{\mathrm{ref}}, r_{\mathrm{endo}})$, computed for each cohort, should not be truncated by the choice of parameter bounds. This was verified by project-

ing Q_{tot} onto each parameter axis, as shown in Fig. 2. The parameter bounds defined by Eq. 1 enclose the Q_{tot} distribution, with the exception of $T_{\text{ref}} = 180$ kPa, an upper bound we imposed in the light of other studies [1, 2].

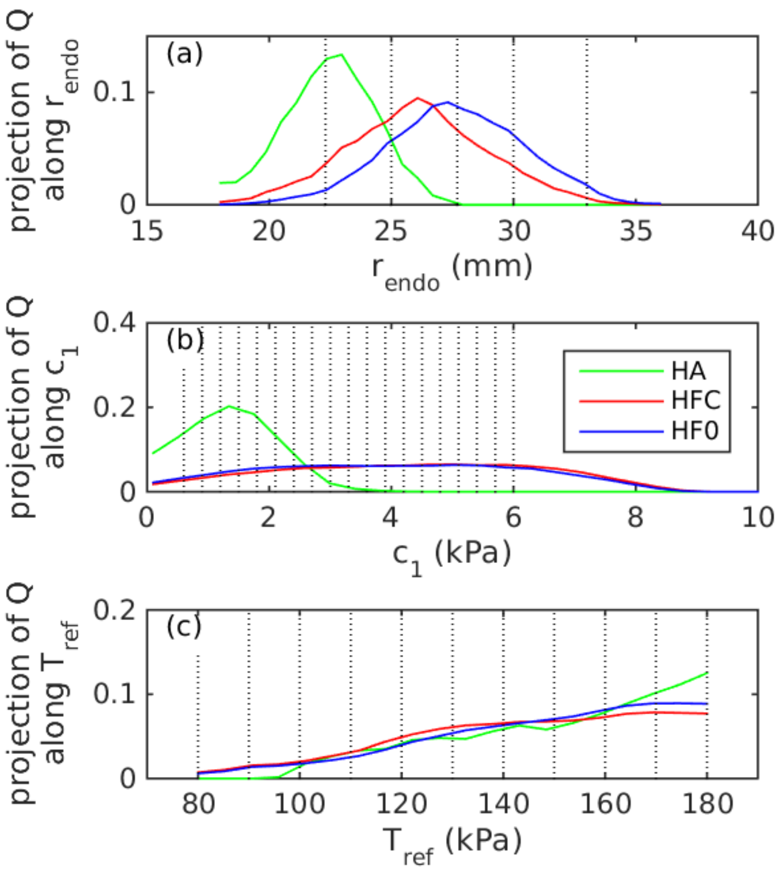

Figure 2. Projections of the three dimensional arrays $Q_{\text{tot}}(c_1, T_{\text{ref}}, r_{\text{endo}})$, obtained from the HA, HF_C, and HF_0 cohort data, onto the (a) r_{endo}, (b) c_1, and (c) T_{ref} axes. The vertical dotted lines indicate the values used in the simulations. The other values were calculated from a trivariate cubic polynomial fittings of each phenotype LVEDD, MEP, and LVEF.

2.3 Confounding factors: further simulations

2.3.1 Gender imbalance in the cohorts

The recruited patient cohorts summarized in Tables S1-S3 show notable differences in gender balance, with the HA controls being predominantly male and the HF_C and HF_0 patients more biased toward females (breast cancer patients). Various gender-dependences of the cardiac system have been reported [3–6]. In particular, both LV mass and LVEDV are on average reportedly 70% the values in males [7]. To test the potential impact of this particular discrepancy on our conclusions, we repeated simulations after scaling the LV meshes to 89% ($0.89 = 0.70^{1/3}$) in all dimensions to represent "female" meshes. The outputs were analysed using these smaller meshes for mapping the HF_C and HF_0 data. The resulting transitions maps, shown in Fig. S3, display no qualitative difference with those in Fig. 5, supporting the conclusion that gender imbalance did not significantly alter our main findings.

2.3.2 LV elongation

Echocardiography measurements were not available to constrain the LV long-axis dimension, and the simulations therefore assumed a constant reference cavity length of 60 mm. To estimate the potential impact of LV elongation in heart failure, we repeated simulations and the analysis after scaling the cavity length in proportion with the endocardial radius r_{endo}. The results, displayed in Fig. S4, show now qualitative difference with the main results, supporting the hypothesis that remodeling of the long-axis dimension in heart failure does not significantly affect our conclusions. Average $\langle m_{\mathrm{rendo}} \rangle$ values were 1.20 and 1.13 for the HF_0 and HF_C transitions, respectively.

2.3.3 Fiber-angle distribution

Some authors have reported a modification of the fiber-orientation distribution in cases of dilated cardiomyopathy and following pressure overload, with fibers becoming more oblique relative to the LV wall cross section, [8, 9]. To assess the potential impact of changes in fiber orientation on our conclusions, we repeated simulations using the "dilated" mesh with $r_{\mathrm{endo}} = 30$ mm, making the endocardial and epicardial fiber directions either more oblique ($+10°$) or more circumferential ($-10°$), relative to the baseline fiber

configuration. The effect on the simulated phenotypes was generally minimal (see Table S6). In particular, the maximum change in LVEF was approximately 3%, significantly less than the > 20% caused by heart failure. This effect arguably does not affect our qualitative conclusions.

Table 6. Sensitivity of simulated phenotypes to the obliqueness α of the muscle fibers. Simulations were repeated for a range of c_1 and T_{ref} values, using the 'inflated' LV mesh with $r_{\mathrm{endo}} = 30$ mm. For each of the phenotypes LVEDD, MEP, and EF, the three columns represent the base line value ($\alpha_{\mathrm{epi}} = -60°$, $\alpha_{\mathrm{endo}} = +80°$ and the changes to this baseline in the case of more circumferential ("+cir": $\alpha_{\mathrm{epi}} = -50°$, $\alpha_{\mathrm{endo}} = +70°$) or more oblique fibers ("+obl": $\alpha_{\mathrm{epi}} = -70°$, $\alpha_{\mathrm{endo}} = +90°$).

c_1	T_{ref}	LVEDD (mm)	+cir	+obl	MEP (mmHg)	+cir	+obl	EF (%)	+cir	+obl
1.5	110	70.6	-0.7	-0.1	124.6	-2.0	+0.1	20.3	-3.2	+0.7
1.5	140	70.4	-0.7	+0.3	136.7	-3.2	-0.2	36.0	-2.9	+1.4
1.5	180	70.3	-0.4	+0.2	148.0	-2.4	+0.9	50.6	-2.5	+0.5
2.4	140	66.6	-0.2	+0.4	130.3	-1.7	+0.2	31.1	-3.2	+1.2
2.4	180	66.6	-0.3	+0.4	140.5	-0.8	+0.0	46.1	-3.1	+1.1
3.3	110	64.7	-0.0	+0.5	117.5	-1.2	+0.2	11.9	-2.5	+1.4
4.2	110	63.8	-0.2	+0.2	117.0	-1.8	-0.2	10.4	-2.1	+0.8
4.2	180	63.9	-0.3	+0.1	135.2	-1.9	-0.1	41.2	-2.0	+0.0
5.1	110	63.3	-0.4	-0.0	116.7	-1.7	-0.5	9.7	-2.4	+0.3
5.1	140	63.4	-0.4	-0.1	125.2	-1.6	+0.2	25.3	-2.4	+0.0
5.1	180	63.4	-0.4	-0.0	134.7	-1.4	+0.3	39.4	-2.3	-0.5
6.0	140	62.4	+0.1	+0.4	123.5	-0.7	+0.8	24.1	-1.8	+0.4
6.0	180	62.3	+0.2	+0.5	133.7	-1.1	-1.5	37.0	-1.1	+1.1

2.3.4 Filling pressure

The LV filling pressure was assumed to be fixed for all the simulations, but is know to increase in HF [10, 11]. This property was however not characterized in our patient cohorts. To estimate the potential sensitivity of our conclusions to this effect, we repeated the simulations by doubling the filling pressure (3.7 mmHg to 7.5 mmHg). As expected, the results display an increase in the stiffness ratio m_{c1} (Fig. 5, but the general qualitative comparison between the $\mathrm{HF_C}$ and $\mathrm{HF_0}$ results remains unchanged, with the former showing a stronger contribution from decreasing T_{ref}.

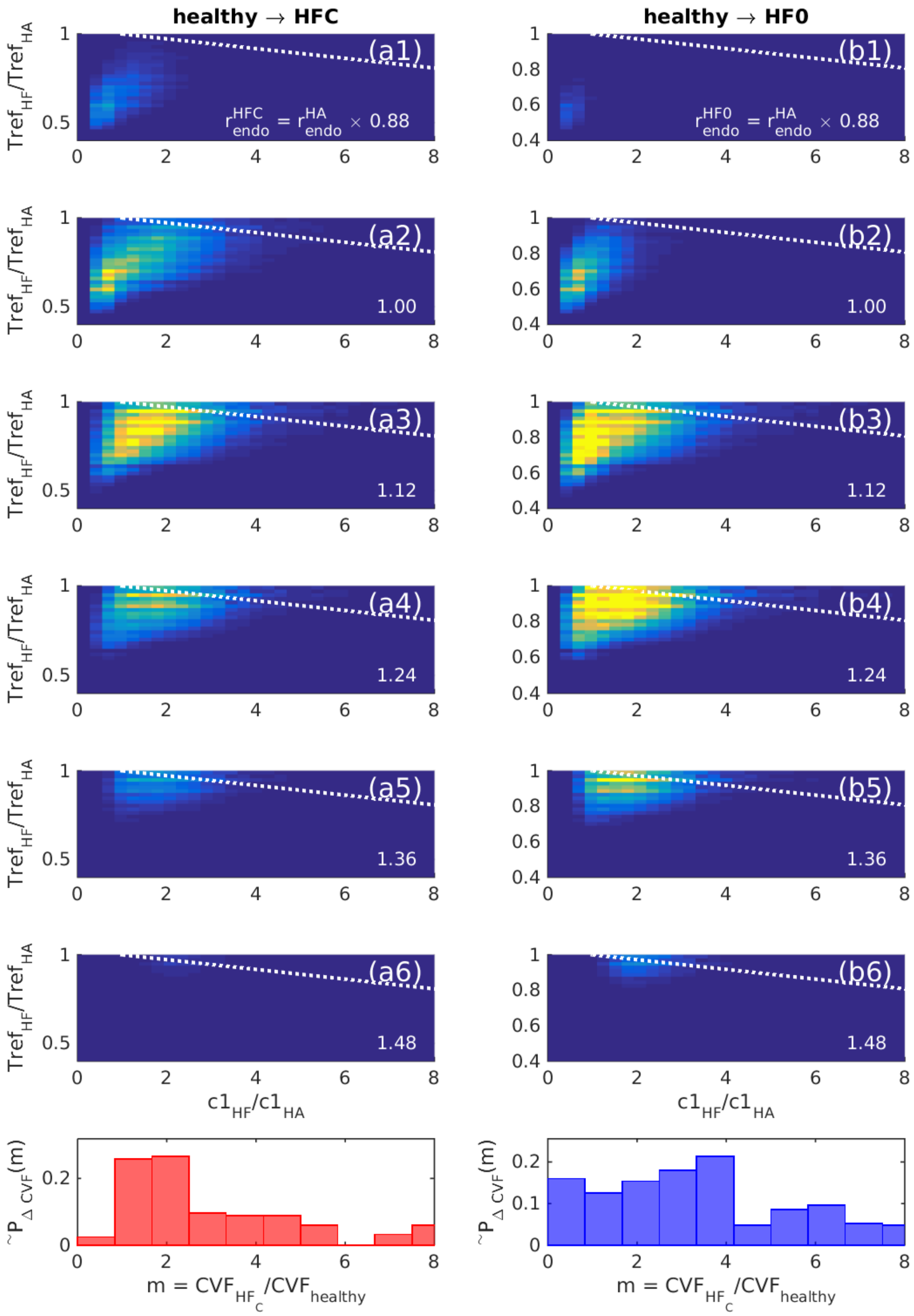

Figure 3. Transition maps representing the parameter transformations from healthy adults to heart-failure patients, represented by smaller "female" LV meshes.

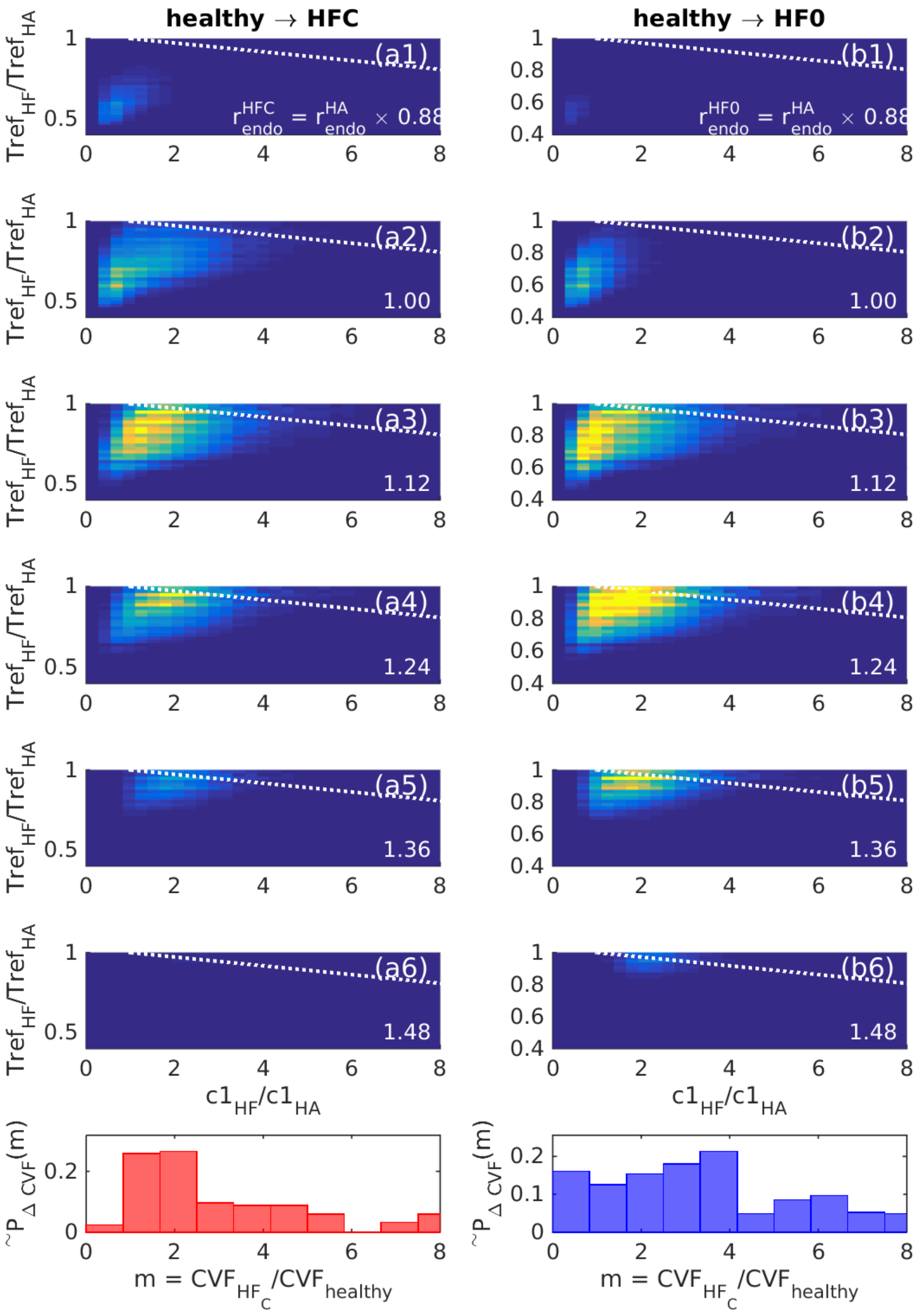

Figure 4. Transition maps representing the parameter transformations from healthy adults to heart-failure patients, assuming a scaling of the ventricular long axis in proportion with the endocardial radius r_{endo}.

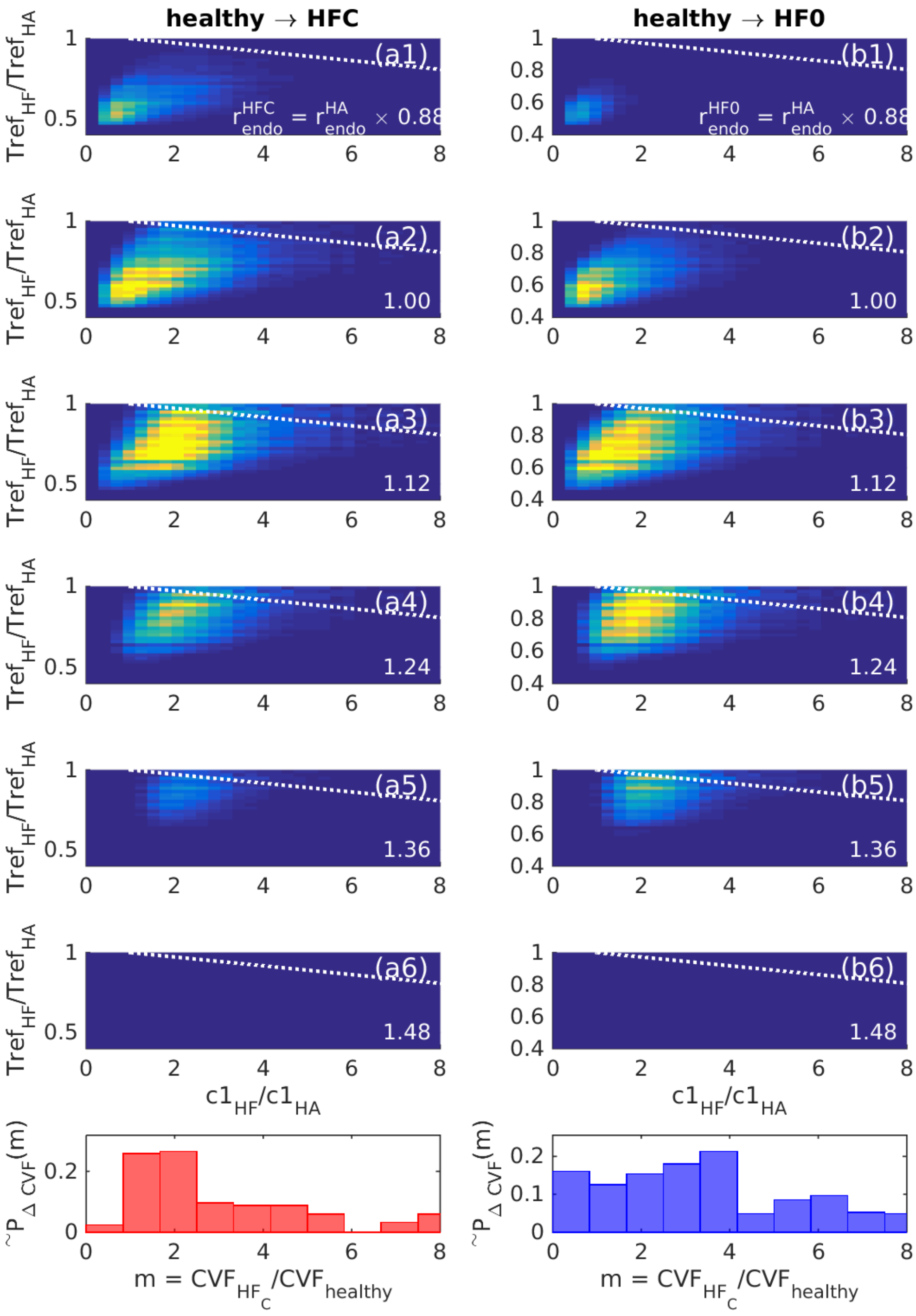

Figure 5. Transition maps representing the parameter transformations from healthy adults to heart-failure patients, assuming a filling pressure of 3.7 mmHg (0.5 kPa) for healthy hearts and 7.5 mmHg (1.0 kPa) for HF.

References

[1] Kenneth S. Campbell. Compliance Accelerates Relaxation in Muscle by Allowing Myosin Heads to Move Relative to Actin. *Biophysical Journal*, 110(3):661–668, 2016.

[2] Sander Land, So Jin Park-Holohan, Nicolas P. Smith, Cristobal G. dos Remedios, Jonathan C. Kentish, and S Niederer. A model of cardiac contraction based on novel measurements of tension development in human cardiomyocytes. *Journal of Molecular and Cellular Cardiology*, 106:68–83, 2017.

[3] Héctor Bidoggia, Juan P. Maciel, Norberto Capalozza, Susana Mosca, Enrique J. Blaksley, Esteban Valverde, Guillermo Bertran, Pedro Arini, Marcelo O. Biagetti, and Ricardo A. Quinteiro. Sex-dependent electrocardiographic pattern of cardiac repolarization. *American Heart Journal*, 140(3):430–436, 2000.

[4] M. Odette Gore, Stephen L. Seliger, Christopher R. Defilippi, Vijay Nambi, Robert H. Christenson, Ibrahim A. Hashim, Ron C. Hoogeveen, Colby R. Ayers, Wensheng Sun, Darren K. McGuire, Christie M. Ballantyne, and James A. De Lemos. Age- and sex-dependent upper reference limits for the high-sensitivity cardiac troponin T assay. *Journal of the American College of Cardiology*, 63(14):1441–1448, 2014.

[5] Thomas H. Fischer, Jonas Herting, Jörg Eiringhaus, Steffen Pabel, Nico H. Hartmann, David Ellenberger, Martin Friedrich, André Renner, Jan Gummert, Lars S. Maier, Markus Zabel, Gerd Hasenfuss, and Samuel Sossalla. Sex-dependent alterations of Ca21 cycling in human cardiac hypertrophy and heart failure. *Europace*, 18(9):1440–1448, 2016.

[6] Babbette LaMarca and Barbara Alexander, editors. *Sex Differences in Cardiovascular Physiology and Pathophysiology.* Academic Press (Elsevier), 2019.

[7] Christine H. Lorenz, Eloisa S. Walker, Victoria L. Morgan, Stacy S. Klein, and Thomas P. Graham. Normal Human Right and Left Ventricular Mass, Systolic Function, and Gender Differences by Cine Magnetic Resonance Imaging. *Journal of Cardiovascular Magnetic Resonance*, 1(1):7–21, 1999.

[8] Michael D. Eggen, Cory M. Swingen, and Paul A. Iaizzo. Analysis of fiber orientation in normal and failing human hearts using diffusion tensor MRI. *Proceedings - 2009*

IEEE International Symposium on Biomedical Imaging: From Nano to Macro, ISBI 2009, pages 642–645, 2009.

[9] T. E. Carew and J. W. Covell. Fiber orientation in hypertrophied canine left ventricle. *American Journal of Physiology-Heart and Circulatory Physiology*, 236(3):H487–H493, 1979.

[10] Michiya Ohno, Che Ping Cheng, and William C. Little. Mechanism of altered patterns of left ventricular filling during the development of congestive heart failure. *Circulation*, 89(5):2241–2250, 1994.

[11] P. W. Armstrong, T. P. Stopps, S. E. Ford, and A. J. De Bold. Rapid ventricular pacing in the dog: Pathophysiologic studies of heart failure. *Circulation*, 74(5):1075–1084, 1986.
